# The Value of Diffusion Tensor Imaging in Differentiating High-Grade Gliomas from Brain Metastases: A Systematic Review and Meta-Analysis

**DOI:** 10.1371/journal.pone.0112550

**Published:** 2014-11-07

**Authors:** Rui Jiang, Fei-Zhou Du, Ci He, Ming Gu, Zhen-Wu Ke, Jian-Hao Li

**Affiliations:** Department of Medical Imaging, Chengdu Military General Hospital, Chengdu, China; University of New Mexico, United States of America

## Abstract

**Purpose:**

Differentiation of high-grade gliomas and solitary brain metastases is an important clinical issue because the treatment strategies differ greatly. Our study aimed to investigate the potential value of diffusion tensor imaging (DTI) in differentiating high-grade gliomas from brain metastases using a meta-analytic approach.

**Materials and Methods:**

We searched Pubmed, Embase and the Cochrane Library for relevant articles published in English. Studies that both investigated high-grade gliomas and brain metastases using DTI were included. Random effect model was used to compare fractional anisotropy (FA) and mean diffusivity (MD) values in the two tumor entities.

**Results:**

Nine studies were included into the meta-analysis. In the peritumoral region, compared with brain metastases, high-grade gliomas had a significant increase of FA (SMD  = 0.47; 95% CI, 0.22–0.71; P<0.01) and a significant decrease of MD (SMD  = −1.49; 95% CI, −1.91 to −1.06; P<0.01). However, in the intratumoral area, no significant change in FA (SMD  = 0.16; 95% CI, −0.49 to 0.82; P = 0.73) or MD (SMD  = 0.34; 95% CI, −0.91 to 1.60; P = 0.59) was detected between gliomas and metastases.

**Conclusions:**

High-grade gliomas may be distinguished from brain metastases by comparing the peritumoral FA and MD values. DTI appears to be a promising tool in diagnosing solitary intracranial lesions.

## Introduction

High-grade gliomas and brain metastases are two of the most common malignant brain tumors in adults. In general, the differentiation between them is possible with clear clinical history or the presence of multiple metastatic lesions. However, it is hard to see the distinction in a patient presenting with solitary metastatic mass and unknown primary malignancy, as these two neoplasms often display similar signal intensity features and contrast enhancement patterns on conventional MR imaging [1].

Recently, diffusion tensor imaging (DTI) has been widely used in intracranial neoplasms. As an advanced MR technique, it describes the movement of water molecules by using two metrics, fractional anisotropy (FA) and mean diffusivity (MD), which represent the directionality and magnitude of water diffusion, respectively [2]. DTI has the advantage of giving more detailed information about the involvement and integrity of the matter tracts in the peritumoral regions [3]. Both primary and metastatic intracranial tumors are surrounded by some degree of hyperintensity on T2-weighted images, which has been recognized as vasogenic edema. Because the edema involves an increased water component, altered DTI metrics have been detected within this surrounding region [2], [4]. Compared with contralateral normal appearing white matter, FA values in lesions of gliomas or metastases have been reported to be consistently reduced [2], [5]–[7]. Further, some authors reported significantly higher FA from the peritumoral region of high-grade gliomas compared with brain metastases [8]–[10]. However, some studies drew diametrically opposite conclusions [2], [5], [11]. Similar controversy existed in the value of MD for discerning gliomas from brain metastases [8], [12].

In view of the conflicting results as well as insufficient previous evidence, we performed this systematic review and meta-analysis, aiming to investigate the potential value of DTI in differentiating high-grade gliomas from solitary brain metastases.

## Methods

### Search Strategy

The overview of this meta-analysis was conducted in accordance with the Preferred Reporting Items for Systematic Reviews and Meta-analysis (PRISMA) statement [13]. A computerized bibliographic search for all relevant articles from 1980 to August 2014 was performed by Pubmed, Embase and the Cochran Library. We used the following search key words: “glioma”, “glioblastoma”, “intracranial neoplasm”, “brain metastases”, “metastatic brain tumor”, “DTI”, and “diffusion tensor”. The language was limited to English. We also manually searched the references of selective articles to identify additional potentially relevant studies.

### Selection Criteria

The inclusion criteria for our meta-analyses were as follows: (1) articles were published in peer-reviewed, English-language journals between January 1980 and August 2014; (2) studies reported DTI metrics in high-grade gliomas (WHO Grade III–IV) and solitary brain metastases, with mean FA or MD values available for effective calculations; (3) the analysis method was a region of interest analysis (ROI), with peritumoral and/or intratumoral regions investigated.

### Data Extraction and Quality Assessment

Two assessors (RJ and MG) independently reviewed the full manuscripts of included studies. Data were extracted in standardized data-collection forms. Extracted data included first author's name; year of publication; study design; region; sample size; patients' mean age; number of participants in each group; DTI invariants; strength of the magnetic field; analysis method; FA and MD values in peritumoral or intratumoral regions. Any discrepancy was resolved by discussion or a third author (FZD). Considering the diagnostic feature of our study, the included articles were critically appraised by the QUADAS tool [14].

### Statistical Analysis

All meta-analyses were performed using the STATA software (version 12.0; Stata Corporation, College Station, Texas). The standardized mean difference (SMD) was calculated and used as the effect-size statistic. The SMD is the standardized difference between two means and can be calculated as the difference between the gliomas and metastases groups divided by the pooled standard deviation (SD). Cohen's pooled SMD was used to characterize the changes. We focused on two major DTI metrics, namely FA and MD. A meta-analysis was performed when more than three studies reported the invariants. The random effect model was employed for all meta-analyses to minimize the potential heterogeneity between studies. Statistical heterogeneity among studies was mainly assessed by the I^2^ statistic. For the I^2^ metric, we considered low, moderate and high I^2^ values to be 25%, 50%, and 75%, respectively [15]. The potential source of heterogeneity was explored by sensitivity and subgroup analyses. To assess whether a specific covariate influenced the effect, meta-regression analysis was conducted by the random-effects model. The covariates of statistical significance were further included into multiple meta-regression. In case of false positive results, the permutation test was carried out to calculate an adjusted P value. The Egger test was used to assess publication bias [16]. A threshold of P<0.1 was used to decide whether heterogeneity or publication bias existed. In other ways, P values were two sided with a significance level of 0.05. Inter-rater reliabilities were calculated by Cohen κ statistics, with 5 levels of agreement, namely poor (κ = 0.00–0.20), fair (κ = 0.21–0.40), moderate (κ = 0.41–0.60), good (κ = 0.61–0.80), and very good (κ = 0.81–1.00) [17].

## Results

### Literature Search

**Figure 1:**
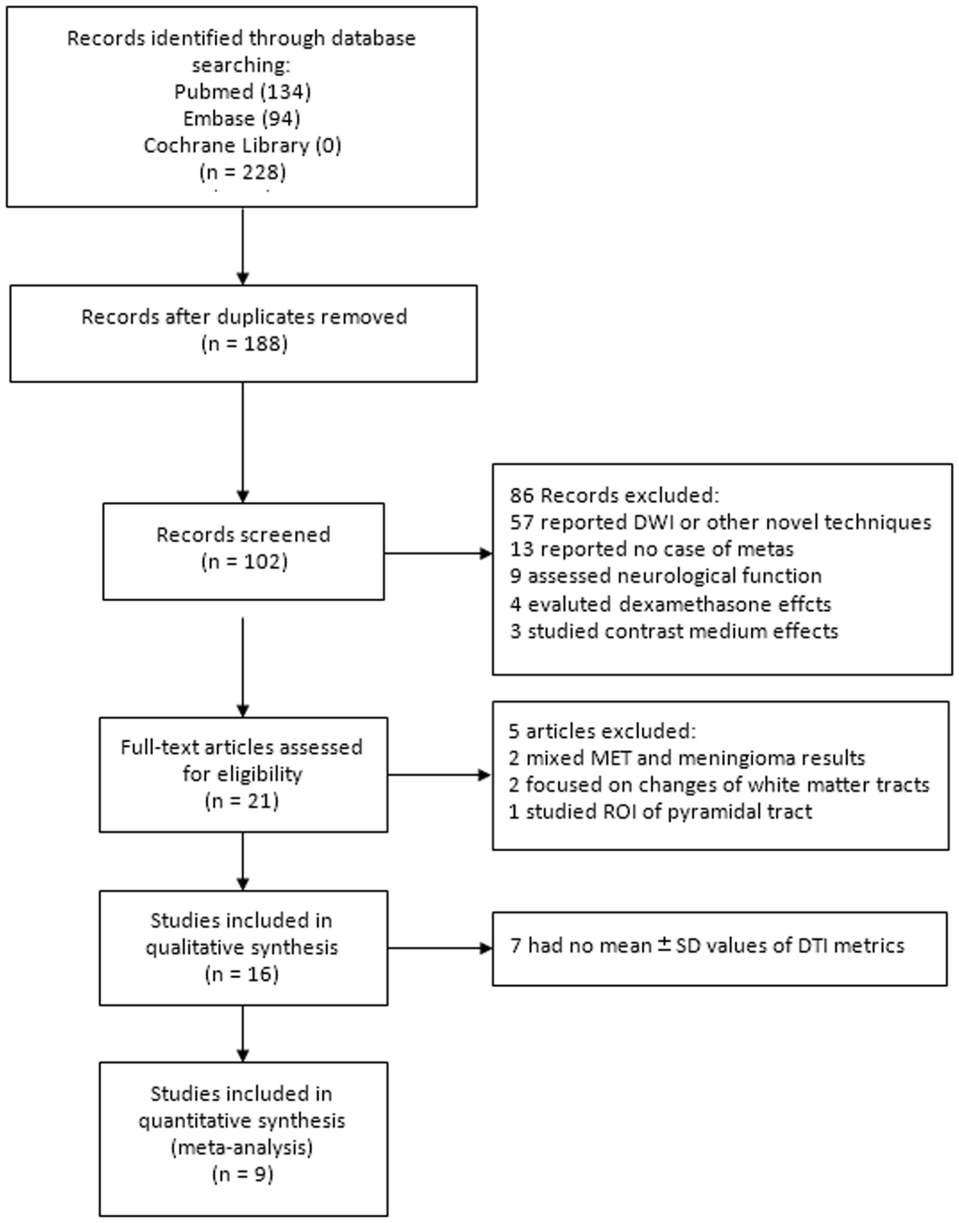
The flowchart shows the selection of eligible studies for meta-analysis.

Our study selection process was shown in Figure 1. The initial search yielded 228 articles, of which 102 studies were selected as potential candidates after screening of titles and abstracts. Fifty-seven studies were excluded because they only reported data from diffusion-weighted imaging or other techniques, but not relating to DTI. Thirteen studies with no case of brain metastases were discarded. Nine studies were excluded because they focused on the evaluation of neurological functions. Seven studies mainly assessing the effects of dexamethasone or contrast medium were discarded. Thus, 21 potentially eligible studies were selected. Five articles were further excluded, including 2 studies with mixed results of meningioma and brain metastases, 2 articles focusing on the change of white matter tracts, and one study only selecting the pyramidal tract as ROI. Sixteen studies were included in qualitative synthesis. As seven studies had no sufficient data to calculate the effect size [8], [9], [11], [18]–[21], nine studies were pooled for meta-analyses. The manual search of reference lists of these articles did not produce any new eligible study. Agreement on selection of studies between two assessors was very good (κ = 0.86).

### Study Characteristics

**Figure 2:**
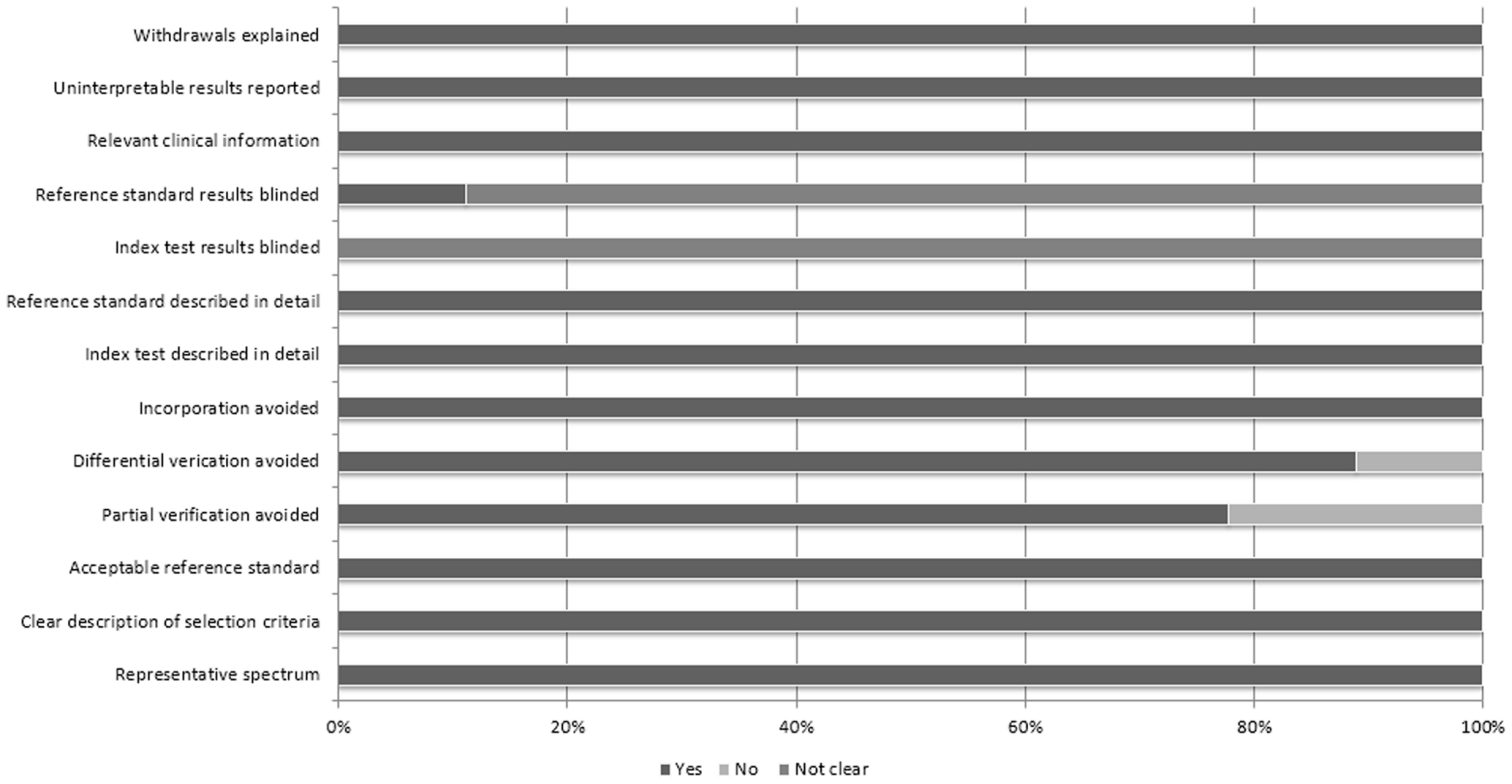
Quality assessement with 13-items modified QUADAS tool.

Nine studies, including three prospective studies and six retrospective studies, were pooled into meta-analyses [2], [5]–[7], [10], [12], [22]–[24]. Totally, 193 patients with high-grade gliomas and 141 patients with solitary brain metastases were involved. Six studies compared glioblastoma (WHO Grade IV) with metastases, and 3 studies compared high-grade gliomas (WHO Grade III–IV) with metastases. The mean age ranged from 50 to 64 years. Six studies utilized a 3.0 tesla scanner and three used a 1.5 tesla scanner. All investigated FA values, whereas only four examined MD values. All reported peritumoral metrics, and six additionally reported intratumoral metrics. The study characteristics were presented in Table 1. Extracted data were displayed in Table S1. Assessed by the 13 items modified QUADAS tool, most studies were of high quality with scores from 10 to 12, which satisfied the majority of standards (Figure 2). Histopathological examination was reference standard in most studies, except for two studies that included metastases participants diagnosed only by MRI [5], [24]. Although DTI was unanimously performed before surgical histopathology confirmation, we cannot preclude the possibility of analyzing DTI results some other time with the knowledge of pathological diagnosis. In fact, only one study stated that the DTI analysis was conducted with such blindness [2]. Similarly, no study stated that the pathological results were obtained without awareness of preoperative MRI reports.

**Table 1:** Characteristics of included studies for meta-analysis.

Author (year)	Sample size	Study design	Location	Mean age, y	Groups (no.)	Field strength, tesla	DTI invariants
Lu (2003)	24	Retrospective	USA	50	HGG (12) vs. MET (12)	1.5	FA, MD
Lu (2004)	20	Retrospective	USA	52	GBM (10) vs. MET (10)	1.5	FA, MD, TII
Tsuchiya (2005)	14	Retrospective	Japan	55	HGG (7) vs. MET (7)	1.5	FA, FA map
Wang (2009)	43	Retrospective	Australia	64	GBM (27) vs. MET (16)	1.5	FA, p, q, L
Byrnes (2010)	28	Prospective	UK	60	GBM (16) vs. MET (12)	1.5	FA, MD
Toh (2011)	41	Retrospective	Taiwan	59	GBM (15) vs. MET (26)	3.0	FA, geometric tensor metrics
Tsougos (2012)	49	Prospective	Greece	NA	GBM (35) vs. MET (14)	3.0	FA
Svolos (2013)	71	Prospective	Greece	NA	HGG (53) vs. MET (18)	3.0	FA
Hoefnagels (2014)	40	Retrospective	Netherland	NA	GBM (18) vs. MET (22)	1.5	FA, MD

FA, fractional anisotropy; GBM, glioblastoma multiforme; HGG, high-grade glioma; MD, mean diffusivity; MET, brain metastases; TII, tumor infiltration index.

### Peritumoral FA

**Figure 3:**
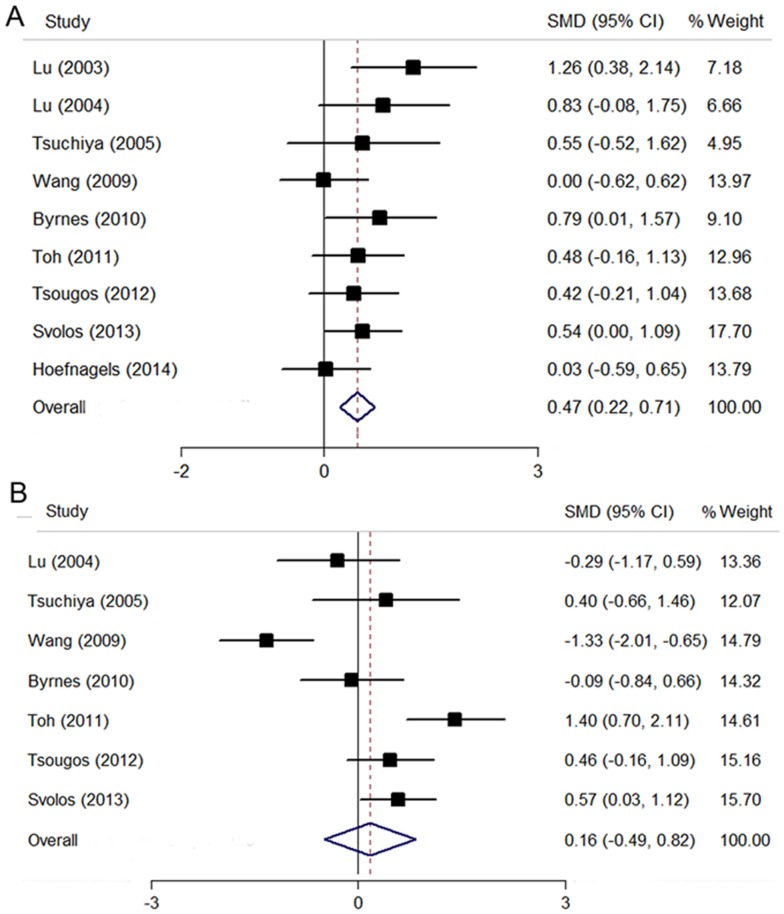
The comparison of FA values between high-grade gliomas and brain metastases. (A) FA values in the peritumoral region. (B) FA values in the intratumoral region.

All nine studies reported value of FA in the peritumoral edema region. The pooling data revealed a significant increase of FA in gliomas compared with brain metastases (SMD  = 0.47; 95% CI, 0.22–0.71; P<0.01), with a low level of heterogeneity (I^2^ = 6.9%; P = 0.38) (Figure 3A). The funnel plot was found to be symmetrical, suggesting a low likelihood of publication bias. No publication bias was revealed by Egger test either (P = 0.12).

The potential sources of heterogeneity were explored by sensitivity and stratifying analyses. No significantly changed result was shown when excluding studies one by one. Subgroups were stratified by study design, glioma types, and filed strength of MRI. We noted that the increase of FA was similarly significant in subgroups (Table 2). The publication year, sample size, field strength, glioma types, and the study region were examined as single covariate in meta-regression analyses. However, none of them showed significant independent effect on the FA values (P>0.1).

**Table 2:** Subgroup analyses for studies investigating FA values in the peritumoral region.

Subgroups	No. of studies	SMD	95% CI	P value	I^2^
Study design					
Prospective	3	0.55	0.19–0.92	0.003	0
Retrospective	6	0.44	0.66–0.82	0.024	34.3
Type of gliomas					
High-grade glioma	3	0.71	0.29–1.13	0.001	0
Glioblastoma	6	0. 35	0.08–0.63	0.012	0
Field strength (T)					
3.0	3	0.49	0.14–0.83	0.006	0
1.5	6	0.50	0.08–0.92	0.019	41.0

### Intratumoral FA

Seven studies were included for pooling FA value in the intratumoral region [5]–[7], [10], [12], [22], [23]. No significant difference was detected between gliomas and brain metastases (SMD  = 0.16; 95% CI, −0.49 to 0.82; P = 0.73), with a high level of heterogeneity (I^2^ = 82.8%; P<0.01) (Figure 3B). The funnel plot was symmetrical, suggesting a low likelihood of publication bias. No publication bias was revealed by Egger test either (P = 0.73).

When exploring the potential sources of heterogeneity by sensitivity analysis, no significant change was shown when excluding studies one by one. In stratified analyses, the increase of FA was significant in subgroups of prospective studies, high-grade gliomas, and field strength of 3.0 tesla (Table 3). In meta-regression of single covariates, including the publication year, sample size, field strength, glioma types, and the study region, only the field strength showed a weak significance (P = 0.06), which may serve as a potential source of heterogeneity.

**Table 3:** Subgroup analyses for studies investigating FA values in the intratumoral region.

Subgroup	No. of studies	SMD	95% CI	P value	I^2^
Study design					
Prospective	3	0.38	0.01–0.75	**0.042**	3.5
Retrospective	4	0.04	−1.26–1.34	0.952	90.2
Type of gliomas					
High-grade glioma	2	0.54	0.05–1.02	**0.030**	0
Glioblastoma	5	0.04	−0.88–0.95	0.940	87.6
Field strength (T)					
3.0	3	0.78	0.24–1.32	**0.005**	55.6
1.5	4	−0.38	−1.13–0.37	0.316	69.4

Significant P values were shown in bold.

### Peritumoral MD

**Figure 4:**
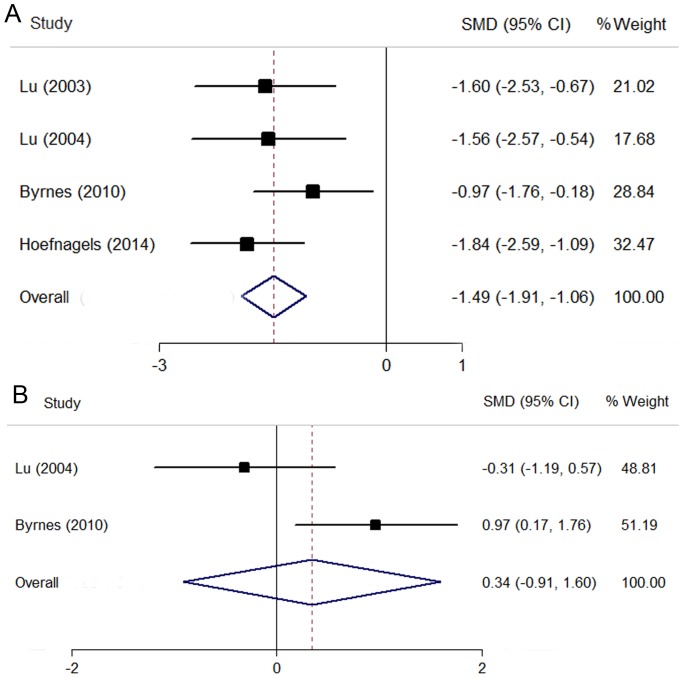
The comparison of MD values between high-grade gliomas and brain metastases. (A) MD values in the peritumoral region. (B) MD values in the intratumoral region.

Four studies reported value of MD in the peritumoral edema region [2], [10], [12], [24]. The pooling data revealed a significant decrease of MD in gliomas compared with brain metastases (SMD  = −1.49; 95% CI, −1.91 to −1.06; P<0.01), with a low level of heterogeneity (I^2^ = 0; P = 0.46) (Figure 4A). The funnel plot was found to be symmetrical, suggesting a low likelihood of publication bias. No publication bias was revealed by further Egger test (P = 0.98). The too few studies precluded sensitivity, subgroup or meta-regression analyses.

### Intratumoral MD

Only two studies were included for pooling MD value in the intratumoral region [10], [12]. No significant difference was detected between gliomas and brain metastases (SMD  = 0.34; 95% CI, −0.91 to 1.60; P = 0.59), with a high level of heterogeneity (I^2^ = 77.6%; P<0.05) (Figure 4B). The too few studies precluded sensitivity, subgroup or meta-regression analyses.

## Discussion

The accurate diagnosis of gliomas and solitary brain metastases is essential to the choice of therapeutic approach and to the assessment of prognosis. However, they often display similar inhomogeneous signal intensity characteristics and irregular contrast enhancement patterns on conventional MR imaging. In recent years, DTI is increasingly being used as a potentially useful tool to investigate brain tumors. To the best of our knowledge, this is the first meta-analysis of DTI studies concerning with the differentiation of these two tumor entities.

Our study demonstrated that FA from the peritumoral regions of high-grade gliomas was significantly higher than that of solitary brain metastases. The difference remained significant in all subgroup analyses. The result may be explained by the different degrees of tumor infiltration in these two tumor types [10]. FA is mainly affected by tumor infiltration [25]. In fact, histological examination has shown that glioblastomas had a greater degree of tumor infiltration within the peritumoral edema region than metastases [4], [10]. In addition, experimental studies demonstrated that glioblastoma cells produced plenty of tumor-specific extracellular matrix components. Serving as substrates for adhesion and subsequent migration of cells through the extracellular space, these molecules accumulated and were oriented in extracellular matrix, leading to a high anisotropy [26].

In contrast with FA results, our study revealed that the MD value was significantly higher in brain metastases than that for high-grade gliomas in the peritumoral region. It may be explained by their different degrees of vasogenic edema [10]. MD is mainly determined by increased extracellular water. Metastatic lesions always had high expression of vascular endothelial growth factor, which greatly helped increase vascular permeability. Thus, a more profound peritumoral vasogenic edema with excessive extracellular fluid was often seen in brain metastases [12]. Notably, the increase in fluid not only led to increased MD but also caused less directionally specific diffusion and in the meantime reduced FA [10].

In the intratumoral region, however, no significant difference was detected between high-grade gliomas and brain metastases when pooling FA or MD data. However, in subgroup of mixed high-grade gliomas types, prospective studies, and studies with field strength of 3.0 tesla, FA from the intratumoral regions of gliomas was significantly higher than that of brain metastases. High-grade gliomas may present higher cellularity in the solid part of tumor than do brain metastases [7], [27], [28]. Further, high-grade gliomas referred to all types of Grade gliomas, including gliomblastomas of course. We inferred that the mixed feature of high-grade gliomas may cause more heterogeneous cellularity compared with gliomblastomas, thus resulting in a significant trend [10]. Nevertheless, only 2 studies with sixty glioma patients were too weak to yield definite conclusion. As is well known, prospective studies are superior to retrospective studies in the control of bias. Compared with 1.5T, 3.0T MRI showed higher signal-to-noise ratio and contrast-to-noise ratio, which allowed better resolution of smaller focal lesions [29]. It has been suggested that DTI at ultra-high field strengths was possible with improved performance in selected ROIs [30]. In addition, only the field strength showed a weak significance in the meta-regression of intratumoral FA results. Thus, we speculated that well-designed researches with high-field DTI may help identify the intratumoral components. However, considering that only three studies were included, these subgroup analyses should be interpreted with caution.

We were aware of the limitations of this meta-analysis. As only articles written in English were included, relevant studies published in non-English language journals may be missed. Notably, articles with statistically significant results were more likely to be published and cited in English language journals. Although no publication bias was detected, the test strength was limited by the small number of studies [16]. Besides, only 3 studies were prospectively designed and most studies had small sample sizes. The subjective assessment and placement of ROIs served as another limitation. The selection of large ROIs may cause the partial volume effect, with pixels from normal appearing white matter included into the peritumoral edema zone. The selection of small ROIs may allow for peritumoral regions containing tumor cells to be excluded from measurements [11]. Unanimously, histological confirmation of the peritumoral region was absent. Although it is not routine procedure, tissue biopsy may help correlate the changes in DTI metrics with the physical measurements of water content and tumor cell density [12], [31]. Furthermore, the use of mean values of DTI metrics in the heterogeneous regions may not always reflect the best indication of anisotropy [6]. Limited by unavailable data, we could not stratify brain metastases by their origination from different primary malignancies. Also, our results may be compromised by the heterogeneous size and location of tumors [6]. Regretfully, the receiver operating characteristic (ROC) or threshold analysis was absent in all studies, which may limit our understanding of the diagnostic accuracy of DTI metrics.

Future attempts are warranted to improve the diagnostic value of DTI. The addition of other MRI techniques, including MR spectroscopy, perfusion imaging, and dynamic susceptibility contrast-enhanced MRI, may provide increasing diagnostic values [7], [23]. The joint analyses of MRI metrics, as well as the introduction of rational model and index may allow higher sensitivity and specificity for tumor diagnosis [8], [12].

## Conclusions

Our findings from this meta-analysis demonstrate a significantly higher FA and lower MD in the peritumoral region of high-grade gliomas compared with solitary brain metastases. The results lend support to the use of FA and MD parameters in differentiating high-grade gliomas from brain metastases. Further researches were required to improve the diagnostic performance of DTI in intracranial tumors.

## Supporting Information

Table S1 **Extracted data of DTI metrics in the peritumoral region and intratumoral region of included studies.**
(DOC)

Checklist S1 **PRISMA checklist.**
(DOC)

Diagram S1 **PRISMA flowchart.**
(DOC)

## References

[1] Lee EJ, Ahn KJ, Lee EK, Lee YS, Kim DB (2013) Potential role of advanced MRI techniques for the peritumoural region in differentiating glioblastoma multiforme and solitary metastatic lesions. Clin Radiol 68: e689–697. 23969153 10.1016/j.crad.2013.06.021

[2] Lu S, Ahn D, Johnson G, Cha S (2003) Peritumoral diffusion tensor imaging of high-grade gliomas and metastatic brain tumors. AJNR Am J Neuroradiol 24: 937–941. 12748097 PMC7975803

[3] Witwer BP, Moftakhar R, Hasan KM, Deshmukh P, Haughton V, et al (2002) Diffusion-tensor imaging of white matter tracts in patients with cerebral neoplasm. J Neurosurg 97: 568–575. 12296640 10.3171/jns.2002.97.3.0568

[4] Sternberg EJ, Lipton ML, Burns J (2014) Utility of diffusion tensor imaging in evaluation of the peritumoral region in patients with primary and metastatic brain tumors. AJNR Am J Neuroradiol 35: 439–444. 24052506 10.3174/ajnr.A3702 PMC7964735

[5] Tsuchiya K, Fujikawa A, Nakajima M, Honya K (2005) Differentiation between solitary brain metastasis and high-grade glioma by diffusion tensor imaging. Br J Radiol 78: 533–537. 15900059 10.1259/bjr/68749637

[6] Wang W, Steward CE, Desmond PM (2009) Diffusion tensor imaging in glioblastoma multiforme and brain metastases: the role of p, q, L, and fractional anisotropy. AJNR Am J Neuroradiol 30: 203–208. 18842762 10.3174/ajnr.A1303 PMC7051698

[7] Tsougos I, Svolos P, Kousi E, Fountas K, Theodorou K, et al (2012) Differentiation of glioblastoma multiforme from metastatic brain tumor using proton magnetic resonance spectroscopy, diffusion and perfusion metrics at 3 T. Cancer Imaging. 12: 423–436. 10.1102/1470-7330.2012.0038 PMC3494384 23108208

[8] Wang S, Kim SJ, Poptani H, Woo JH, Mohan S, et al (2014) Diagnostic utility of diffusion tensor imaging in differentiating glioblastomas from brain metastases. AJNR Am J Neuroradiol 35: 928–934. 24503556 10.3174/ajnr.A3871 PMC7964538

[9] Wang S, Kim S, Chawla S, Wolf RL, Zhang WG, et al (2009) Differentiation between glioblastomas and solitary brain metastases using diffusion tensor imaging. Neuroimage 44: 653–660. 18951985 10.1016/j.neuroimage.2008.09.027 PMC2655208

[10] Byrnes TJ, Barrick TR, Bell BA, Clark CA (2011) Diffusion tensor imaging discriminates between glioblastoma and cerebral metastases in vivo. NMR Biomed 24: 54–60. 20665905 10.1002/nbm.1555

[11] van Westen D, Latt J, Englund E, Brockstedt S, Larsson EM (2006) Tumor extension in high-grade gliomas assessed with diffusion magnetic resonance imaging: values and lesion-to-brain ratios of apparent diffusion coefficient and fractional anisotropy. Acta Radiol 47: 311–319. 16613314 10.1080/02841850500539058

[12] Lu S, Ahn D, Johnson G, Law M, Zagzag D, et al (2004) Diffusion-tensor MR imaging of intracranial neoplasia and associated peritumoral edema: introduction of the tumor infiltration index. Radiology 232: 221–228. 15220505 10.1148/radiol.2321030653

[13] Moher D, Liberati A, Tetzlaff J, Altman DG (2009) Group P (2009) Preferred reporting items for systematic reviews and meta-analyses: the PRISMA statement. PLoS Med 6: e1000097. 19621072 10.1371/journal.pmed.1000097 PMC2707599

[14] Whiting P, Rutjes AW, Reitsma JB, Bossuyt PM, Kleijnen J (2003) The development of QUADAS: a tool for the quality assessment of studies of diagnostic accuracy included in systematic reviews. BMC Med Res Methodol 3: 25. 14606960 10.1186/1471-2288-3-25 PMC305345

[15] Higgins JP, Thompson SG, Deeks JJ, Altman DG (2003) Measuring inconsistency in meta-analyses. BMJ 327: 557–560. 12958120 10.1136/bmj.327.7414.557 PMC192859

[16] Egger M, Davey Smith G, Schneider M, Minder C (1997) Bias in meta-analysis detected by a simple, graphical test. BMJ 315: 629–634. 9310563 10.1136/bmj.315.7109.629 PMC2127453

[17] Landis JR, Koch GG (1977) The measurement of observer agreement for categorical data. Biometrics 33: 159–174. 843571

[18] Saksena S, Jain R, Schultz L, Jiang Q, Soltanian-Zadeh H, et al (2013) The Corpus Callosum Wallerian Degeneration in the Unilateral Brain Tumors: Evaluation with Diffusion Tensor Imaging (DTI). J Clin Diagn Res 7: 320–325. 23543618 10.7860/JCDR/2013/4491.2757 PMC3592302

[19] Chen R, Wang S, Poptani H, Melhem ER, Herskovits EH (2013) A Bayesian diagnostic system to differentiate glioblastomas from solitary brain metastases. Neuroradiol J 26: 175–183. 23859240 10.1177/197140091302600207 PMC5228726

[20] Wang S, Kim S, Chawla S, Wolf RL, Knipp DE, et al (2011) Differentiation between glioblastomas, solitary brain metastases, and primary cerebral lymphomas using diffusion tensor and dynamic susceptibility contrast-enhanced MR imaging. AJNR Am J Neuroradiol 32: 507–514. 21330399 10.3174/ajnr.A2333 PMC8013110

[21] Yang G, Jones TL, Barrick TR, Howe FA (2014) Discrimination between glioblastoma multiforme and solitary metastasis using morphological features derived from the p:q tensor decomposition of diffusion tensor imaging. NMR Biomed 27: 1103–1111. 25066520 10.1002/nbm.3163

[22] Toh CH, Wei KC, Ng SH, Wan YL, Lin CP, et al (2011) Differentiation of brain abscesses from necrotic glioblastomas and cystic metastatic brain tumors with diffusion tensor imaging. AJNR Am J Neuroradiol 32: 1646–1651. 21835939 10.3174/ajnr.A2581 PMC7965370

[23] Svolos P, Tsolaki E, Kapsalaki E, Theodorou K, Fountas K, et al (2013) Investigating brain tumor differentiation with diffusion and perfusion metrics at 3T MRI using pattern recognition techniques. Magn Reson Imaging 31: 1567–1577. 23906533 10.1016/j.mri.2013.06.010

[24] Hoefnagels FW, De Witt Hamer P, Sanz-Arigita E, Idema S, Kuijer JP, et al (2014) Differentiation of edema and glioma infiltration: proposal of a DTI-based probability map. J Neurooncol 120: 187–198. 25079117 10.1007/s11060-014-1544-9

[25] White ML, Zhang Y, Yu F, Jaffar Kazmi SA (2011) Diffusion tensor MR imaging of cerebral gliomas: evaluating fractional anisotropy characteristics. AJNR Am J Neuroradiol 32: 374–381. 20947645 10.3174/ajnr.A2267 PMC7965729

[26] Zamecnik J (2005) The extracellular space and matrix of gliomas. Acta Neuropathol 110: 435–442. 16175354 10.1007/s00401-005-1078-5

[27] Kinoshita M, Hashimoto N, Goto T, Kagawa N, Kishima H, et al (2008) Fractional anisotropy and tumor cell density of the tumor core show positive correlation in diffusion tensor magnetic resonance imaging of malignant brain tumors. Neuroimage 43: 29–35. 18672074 10.1016/j.neuroimage.2008.06.041

[28] Rees JH, Smirniotopoulos JG, Jones RV, Wong K (1996) Glioblastoma multiforme: radiologic-pathologic correlation. Radiographics 16: 1413–1438 quiz 1462–1413. 8946545 10.1148/radiographics.16.6.8946545

[29] Zijlmans M, de Kort GA, Witkamp TD, Huiskamp GM, Seppenwoolde JH, et al (2009) 3T versus 1.5T phased-array MRI in the presurgical work-up of patients with partial epilepsy of uncertain focus. J Magn Reson Imaging 30: 256–262. 19629993 10.1002/jmri.21811

[30] Polders DL, Leemans A, Hendrikse J, Donahue MJ, Luijten PR, et al (2011) Signal to noise ratio and uncertainty in diffusion tensor imaging at 1.5, 3.0, and 7.0 Tesla. J Magn Reson Imaging 33: 1456–1463. 21591016 10.1002/jmri.22554

[31] Price SJ, Burnet NG, Donovan T, Green HA, Pena A, et al (2003) Diffusion tensor imaging of brain tumours at 3T: a potential tool for assessing white matter tract invasion? Clin Radiol 58: 455–462. 12788314 10.1016/s0009-9260(03)00115-6

