# Supplementary material for: The Value of Diffusion Tensor Imaging in Differentiating High-Grade Gliomas from Brain Metastases: A Systematic Review and Meta-Analysis
Source: PLoS One. 2014 Nov 7;9(11):e112550. doi: 10.1371/journal.pone.0112550 (PMC4224505; doi:10.1371/journal.pone.0112550)
Supplement: Table S1 — Extracted data of DTI metrics in the peritumoral region and intratumoral region of included studies. (DOC) [file pone.0112550.s001.doc]

**Table S1. Extracted data of DTI metrics in the peritumoral and intratumoral regions** in included studies.

| Author (year) | Regions | HGG FA, mean±SD | HGG MD (10^-3^ mm^2^/s), mean±SD | MET FA, mean±SD | MET MD (10^-3^ mm^2^/s), mean±SD |
| --- | --- | --- | --- | --- | --- |
| Lu (2003) | Peritumoral | 0.248±0.0630 | 0.622±0.111 | 0.181±0.041 | 0.798±0.109 |
| Lu (2004) | Intratumoral | 0.205±0.043 | 0.619±0.099 | 0.226±0.092 | 0.659±0.152 |
|  | Peritumoral | 0.243±0.043 | 0.604±0.100 | 0.211±0.033 | 0.733±0.061 |
| Tsuchiya (2005) | Intratumoral | 0.160±0.050 | NA | 0.140±0.050 | NA |
|  | Peritumoral | 0.200±0.090 | NA | 0.160±0.050 | NA |
| Wang (2009) | Intratumoral | 0.310±0.050 | NA | 0.420±0.120 | NA |
|  | Peritumoral | 0.320±0.050 | NA | 0.320±0.080 | NA |
| Byrnes (2010) | Intratumoral | 0.136±0.021 | 1.220±0.284 | 0.139±0.044 | 0.980±0.188 |
|  | Peritumoral | 0.188±0.045 | 1.250±0.201 | 0.159±0.020 | 1.410±0.097 |
| Toh (2011) | Intratumoral | 0.062±0.017 | NA | 0.047±0.004 | NA |
|  | Peritumoral | 0.171±0.038 | NA | 0.153±0.037 | NA |
| Tsougos (2012) | Intratumoral | 0.147±0.065 | NA | 0.119±0.047 | NA |
|  | Peritumoral | 0.291±0.075 | NA | 0.261±0.063 | NA |
| Svolos (2013) | Intratumoral | 0.148±0.058 | NA | 0.117±0.040 | NA |
|  | Peritumoral | 0.286±0.069 | NA | 0.251±0.048 | NA |
| Hoefnagels (2014) | Peritumoral | 0.195±0.024 | 0.133±0.001 | 0.194±0.039 | 0.136±0.002 |

FA, fractional anisotropy; HGG, high-grade glioma; MD, mean diffusivity; mean±SD, means and standard deviation; MET, brain metastases; NA, not available.
