# Supplementary material for: The Value of Diffusion Tensor Imaging in Differentiating High-Grade Gliomas from Brain Metastases: A Systematic Review and Meta-Analysis
Source: PLoS One. 2014 Nov 7;9(11):e112550. doi: 10.1371/journal.pone.0112550 (PMC4224505; doi:10.1371/journal.pone.0112550)
Supplement: Diagram S1 — PRISMA flowchart. (DOC) [file pone.0112550.s003.doc]

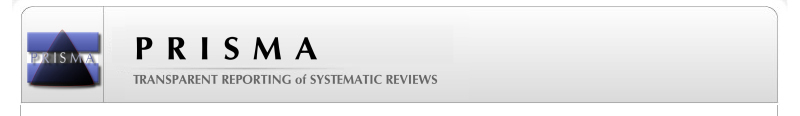
**PRISMA 2009 Flow Diagram**

**Screening**

**Included**

**Eligibility**

**Identification**

Records identified through database searching:

Pubmed (134)

Embase (94)

Cochrane Library (0)

(n = 228)
(n = )

Records after duplicates removed
(n = 188)

Records screened
(n = 102)

86 Records excluded:

57 reported DWI or other novel techniques

13 reported no case of metas

9 assessed neurological function

4 evaluted dexamethasone effcts

3 studied contrast medium effects

Full-text articles assessed for eligibility
(n = 21)

5 articles excluded:

2 mixed MET and meningioma results

2 focused on changes of white matter tracts

1 studied ROI of pyramidal tract

Studies included in qualitative synthesis
(n = 16)

Studies included in quantitative synthesis (meta-analysis)
(n = 9)

7 had no mean±SD values of DTI metrics
